# Cytosolic Sodium Influx in Mesophyll Protoplasts of *Arabidopsis thaliana*, wt, *sos1:1* and *nhx1* Differs and Induces Different Calcium Changes

**DOI:** 10.3390/plants11243439

**Published:** 2022-12-09

**Authors:** Sherif H. Morgan, Md Abdul Kader, Sylvia Lindberg

**Affiliations:** 1 Plant Botany Department, Faculty of Agriculture, Cairo University, Cairo 12613, Egypt; 2 Department of Agronomy, Bangladesh Agricultural University, Mymensingh 2202, Bangladesh; 3 Department of Ecology, Environment and Plant Sciences, Stockholm University, SE-106 91 Stockholm, Sweden

**Keywords:** *Arabidopsis*, cytosolic Ca^2+^ and Na^+^, epifluorescence ratio microscopy, influx, salt stress

## Abstract

The sodium influx into the cytosol of mesophyll protoplasts from *Arabidopsis thaliana* cv. Columbia, wild type, was compared with the influx into *sos1-1* and *nhx1* genotypes, which lack the Na^+^/H^+^ antiporter in the plasma membrane and tonoplast, respectively. Changes in cytosolic sodium and calcium concentrations upon a 100 mM NaCl addition were detected by use of epifluorescence microscopy and the sodium-specific fluorescent dye SBFI, AM, and calcium sensitive Fura 2, AM, respectively. There was a smaller and mainly transient influx of Na^+^ in the cytosol of the wild type compared with the *sos1-1* and *nhx1* genotypes, in which the influx lasted for a longer time. Sodium chloride addition to the protoplasts’ medium induced a significant increase in cytosolic calcium concentration in the wild type at 1.0 mM external calcium, and to a lesser extent in *nhx1*, however, it was negligible in the *sos1-1* genotype. LiCl inhibited the cytosolic calcium elevation in the wild type. The results suggest that the salt-induced calcium elevation in the cytosol of mesophyll cells depends on an influx from both internal and external stores and occurs in the presence of an intact Na^+^/H^+^ antiporter at the plasma membrane. The *Arabidopsis* SOS1 more effectively regulates sodium homeostasis than NHX1.

## 1. Introduction

### 1.1. Sodium Uptake and Transport

Salinity stress is one of the main factors that restricts crop productivity and has a great impact on economies worldwide [1]. Soil salinity inhibits plant growth; uptake of water, K^+^, and Ca^2+^; and causes ionic imbalance, toxicity, and changed metabolism [2]. Different transporters mediate the uptake of Na^+^ into plant cells, such as nonselective cation channels; NSCCs, which are of two types: cyclic nucleotide-gated channels and CNGCs; and glutamate receptors, GLRs, high-affinity potassium channels, HKTs, and HAKs, which all have been identified in *Arabidopsis* [3,4]. 

Plants have developed several mechanisms to overcome stress and keep ionic homeostasis. Early reports stated that most important for ionic homeostasis are the Na^+^/H^+^ antiporters that transport Na^+^ out of the cell by the Salt-Overly Sensitive pathway, SOS, at the plasma membrane, and NHX antiporters at the tonoplast and other endomembranes that compartmentalize Na^+^ into the vacuole and subcellular organelles [5]. In halophytes with bladder cells, SOS is also involved in salt secretion from the leaves [6]. 

Aside from its role in Na^+^ transport from the cytosol, NHX has a function in K^+^ homeostasis, endosomal pH control, and vesicle trafficking [7]. 

It was reported that the overexpression of *AtNHX1,* and of the wheat Na^+^/H^+^ antiporter *TaNHX*, increases both salt and drought stress tolerance [8,9]. The NHX antiporters mostly are energized by both V-ATPase and V-PPase in the tonoplast, but in *Arabidopsis*, only the V-PPase is involved in Na^+^ transport into the vacuole [10]. However, it has been questioned if the NHX at the tonoplast really transports Na^+^. A possibility is that it instead transports K^+^ for osmotic and stomata regulation [11]. Some reports demonstrate that intracellular vesicle transport and autophagy are important transport mechanisms for Na^+^ [12,13]. 

Results showed that a mutation of AtSOS1, *Atsos1-1,* induced a significant decrease in the transcripts of AtHKT1;1, AtSKOR, and AtAKT1 in the roots compared with Wt, which led to an accumulation of Na^+^ in the roots and a decrease in K^+^ uptake and translocation [14]. The AtHKT1;1 mutation changed transcripts of AtSKOR and AtHAK5 and treatment with 25 mM Na^+^ caused a decrease in the selective transport of K^+^ over Na^+^ in the *Athkt1;1* roots compared with the Wt roots. In rice, the OsHKT1;5 was involved in the transport and regulation of K^+^ and Ca^2+^ homeostasis [15]. These findings show that the transport system under salinity stress is very complex when considering net ion uptake and translocation between root and shoot.

### 1.2. Calcium Transport and Signalling under Salinity

Calcium has a crucial signalling function in salt stress and tolerance. Changes in the cytosolic calcium concentration, [Ca^2+^]_cyt_ depend both on the influx and efflux of Ca^2+^. High salt causes both ionic and osmotic stresses, which induce different Ca^2+^ signalling “signatures” and the production of reactive oxygen species, ROS. Mutation of the ROS-producing NADPH oxidase, RBOHD, slows down calcium signalling [16]. An increase in [Ca^2+^]_cyt_, can occur by calcium transport from the apoplast via plasma membrane channels or from internal calcium stores, such as the ER or vacuoles [17]. In *Arabidopsis*, under salt stress calcium is transported into the cytosol by different channels, such as cyclic nucleotide-gated channels, AtCNGCs, and glutamate receptor-like receptors, AtGLRs, in the plasma membrane ([18] and references therein). Wang et al., (2019) showed that the At GLR_3-7_ interacted with 14-3-3 proteins in the regulation of Ca^2+^_cyt_ under salt stress [14]. Transport of Ca^2+^ from the vacuole into cytosol can be involved in calcium signalling by the two-pore channel, TPC1 in the tonoplast [19]. In *Arabidopsis*, roots ROS are built in the endosomes and then are transported to the central vacuole where they activate Ca-permeable channels [20]. 

In the cytosol of roots, Ca^2+^ can bind to the SOS3/CBL4 protein and the complex in turn activates the serine/threonine kinase, SOS2/CIPK24. This protein is transported to the plasma membrane where it phosphorylates SOS1 with subsequent activation of this protein [21,22]. The phosphorylation relieves SOS1 from its autoinhibitory domain in the C-terminal [23]. The former authors stated that the *sos1-1* mutant has restricted growth compared with the wild type of *Arabidopsis,* which can remain green after treatment with 100 mM NaCl. In the *sos1-1* mutant, the young leaves turned chlorotic and the old leaves became dark. 

In root cells, the putative sensor for calcium, SOS3, is a calcineurin-binding like 10 protein. In *Arabidopsis*, it is called AtCBL10 and located at the tonoplast. However, in shoots, a related protein is proposed to function in a similar way, the SCABP8, an SOS3-like calcium-binding protein 8 [24,25].

Calcium has a signalling function under stress. Salinity stress in plants mainly induces a transient or prolonged elevation of cytosolic calcium concentration, [Ca^2+^_cyt_] [26,27]. The increase in [Ca^2+^_cyt_] activates protein kinases via calmodulin, which in turn induces different cellular reactions leading to reduced stress [28]. 

A report showed that the *Arabidopsis sos1* mutant accumulated less total Na^+^ than the wild type after cultivation in the presence of a low concentration of NaCl (20–25 mM) [29]. In the same report, it was demonstrated that the total Na^+^ accumulation in *Arabidopsis* wild type and *nhx1* mutant did not differ [30]. Since most investigations on salt uptake and tolerance mechanisms deal with gene expressions and total uptake/transport in roots, we studied the cytosolic uptake of Na^+^ into mesophyll cells of *Arabidopsis* when the external concentration of NaCl was high, 100 mM.

In the *Atsos1-1* mutant, the accumulated Na^+^ cannot be transported out of the cell by any SOS1-1 transporter. We hypothesized that the lack of this Na^+^/H^+^ antiporter should result in a higher net [Na^+^]_cyt_ compared to Wt. More Na^+^ should enter into the cytosol in the presence of 0.1 mM Ca^2+^ than at 1.0 mM Ca^2+^, as the latter concentration can block the entrance of Na^+^ by NSCCs and is supposed to function as the main Na^+^ -uptake channels at high salinity [4,31,32].

We also hypothesized that the *nhx1* protoplasts accumulated a higher net [Na^+^] in the cytosol than the Wt cells, as they are lacking transport into the vacuoles or endomembrane vesicles by the NHX1. 

To get better knowledge about the efficiency of SOS1-1 and NHX1 to exclude Na^+^ from the cytosol in *Arabidopsis* mesophyll cells, we measured and compared the kinetic patterns for the net accumulation of Na^+^ in the cytosol of *Arabidopsis* wild type, *sos1-1,* and *nhx1* mutants. To investigate calcium signalling in *Arabidopsis* under salinity we compared the cytosolic [Ca^2+^] elevation of a 100 mM Na^+^ addition to mesophyll protoplasts from *Arabidopsis* wild type, *sos1-1*, and *nhx1* mutants in the presence of different external Ca^2+^ concentrations. LiCl was used to investigate if an IP_3_-regulated Ca^2+^ influx from internal stores took part in the signalling [33].

## 2. Results

### 2.1. Sodium Uptake in Mesophyll Protoplasts

**Figure 1:**
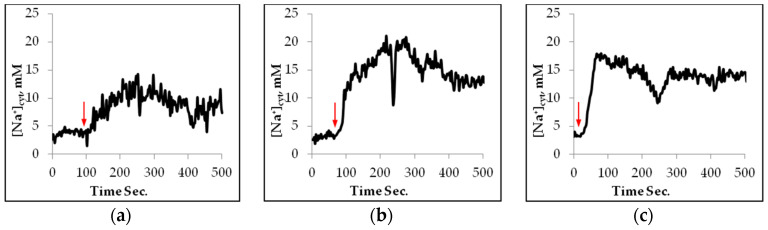
Changes of [Na^+^_cyt_] in mM with time upon addition of 100 mM of NaCl to single mesophyll protoplasts of *Arabidopsis* wild type (**a**), *sos1-1* (**b**), and *nhx1* (**c**). Measurements at excitation ratio 340/380 nm and emission 510–550 nm. Typical traces.

When the seedlings were harvested after 7–8 weeks, the mutants looked somewhat smaller than the wild type. In order to measure any influx of sodium into the mesophyll protoplasts of *Arabidopsis* wild type, *sos1-1,* and *nhx1,* protoplasts were loaded with the sodium-specific dye SBFI AM. Only protoplasts correctly loaded with the dye in the cytosol were used for the experiments. The addition of 100 mM NaCl to the Wt protoplasts in the presence of 1 mM Ca^2+^ caused a transient increase in the fluorescence intensity ratio of 340/380 nm, corresponding to a rather low influx of Na^+^ when the influx stabilized (Figure 1a).

**Figure 2:**
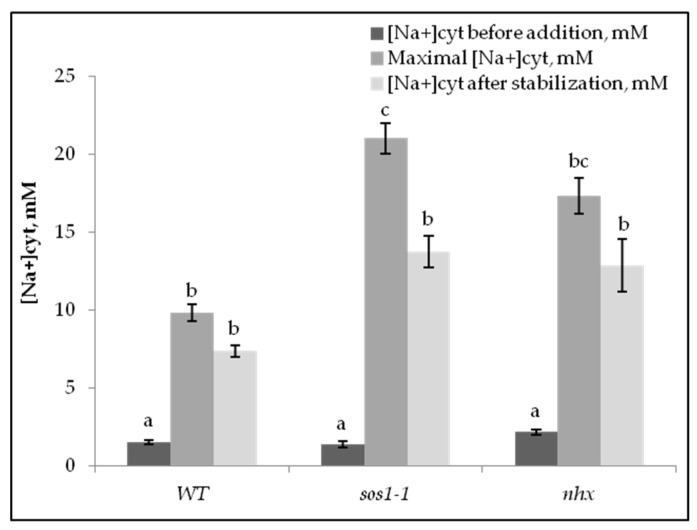
Changes of [Na^+^_cyt ]_ in mM of mesophyll protoplasts of *Arabidopsis* wild type, *sos1-1,* and *nhx1* genotypes upon addition of 100 mM of NaCl, and after stabilization. Means ± SE.

When 100 mM of NaCl instead was added to the genotypes *sos1-1* and *nhx1* protoplasts, the cytosolic influx of sodium at the same external concentration of Ca^2+^ was higher than in the Wt and also remained at a higher level (Figure 1a–c and Figure 2). The maximal influx was obtained in *sos1-1,* and after stabilization, the highest net uptake of Na^+^ was seen in the *sos1-1* mutant. In that mutant, the influx was rapidly increasing after the addition of Na^+^. In the *nhx1* mutant, the increase of [Na^+^]_cyt_ was almost transient. Thereafter it increased and was stable at the end of the experiment for more than 200 s.

### 2.2. Calcium Changes in Mesophyll Protoplasts upon NaCl Addition

**Figure 3:**
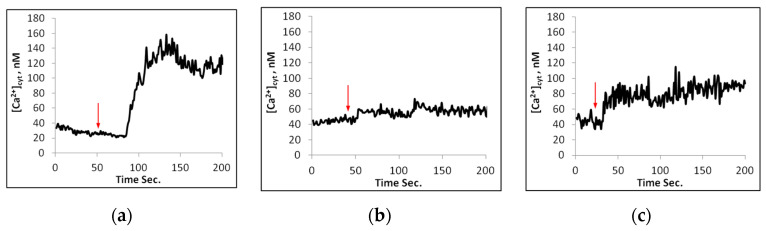
Changes of [Ca^2+^_cyt_] in nM with time, upon addition of 100 mM of NaCl to single mesophyll protoplasts of *Arabidopsis* wild type (**a**), *sos1-1* (**b**), and *nhx1* (**c**). Measurements at excitation ratio 340/380 nm and emission 510–550 nm. Typical traces.

**Figure 4:**
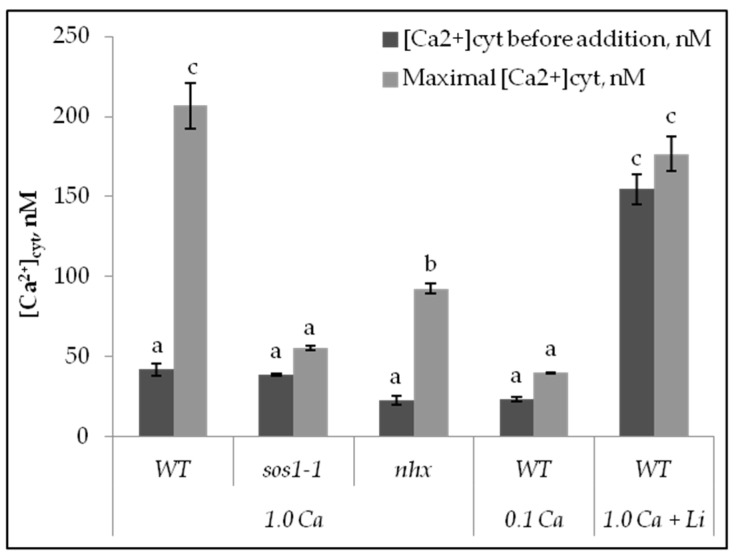
Changes in [Ca^2+^_cyt_] in nM of mesophyll protoplasts of *Arabidopsis* wild type, *sos1-1,* and *nhx1* genotypes before the addition of 100 mM of NaCl and at maximal changes of [Ca^2+^_cyt_]. The external solution was with and without 5 mM LiCl and contained 0.1 or 1.0 mM Ca^2+^. Means ± SE.

For cytosolic calcium Ca^2+^_cyt_ measurements, the protoplasts were loaded with Fura 2, AM, a Ca^2+^-specific fluorescent dye. Upon addition of 100 mM NaCl to the wild-type protoplasts in the presence of 1 mM Ca, there was a distinct increase in cytosolic calcium concentration, [Ca^2^]_cyt_ (Figure 3a and Figure 4). Pretreatment of the protoplasts for 15 min in the presence of 5 mM LiCl significantly decreased the calcium elevation. The maximal increase in [Ca^2^]_cyt_ was obtained in Wt protoplasts in the presence of 1.0 mM external Ca^2+^. At 0.1 mM external Ca^2+^, the elevation of Ca^2^_cyt_ was reduced (Figure 4).

When 100 mM NaCl instead was added to *sos1-1* protoplasts in the presence of 0.1 or 1 mM Ca^2^, there were negligible increases of [Ca^2^]_cyt_ (Figure 3b and Figure 4). On the other hand, when NaCl was added to the *nhx1* protoplasts in the presence of 1 mM Ca^2+^, there was an elevation of [Ca^2^]_cyt_ which was less pronounced than in Wt (Figure 3c and Figure 4).

## 3. Discussion

In the present study, sodium influx into the mesophyll protoplasts was low in the presence of 1.0 mM calcium. Therefore, in *Arabidopsis* Na^+^ is probably mainly taken up by nonselective cation channels, NSCCs, which are permeable for both Ca^2+^ and Na^+^. Sodium can also be taken up by HKT channels as shown in experiments with rice [31]. The high-affinity transporters HKT2s can mediate Na^+^-K^+^ cotransport and Na/K homeostasis under K-starved conditions and salinity [32]. In barley, over-expression of HKT2 caused salt tolerance, however, in *Arabidopsis* HKT2, expression showed reduced salt tolerance.

The importance of nonselective cation channels for the influx in the presence of a high concentration of sodium has been stressed [31,34]. 

A higher influx of Na^+^ was obtained in the *sos1-1* and *nhx1* mutants than in the wild type (Figure 1a–c and Figure 2). The influx into the *sos1-1* protoplasts was faster than in the other genotypes and may depend on the lack of an SOS1 protein, and then decreased due to efflux from the cytosol into the vacuole or intracellular vesicles (Figure 1b). It was reported that SOS1 plays a crucial role in sodium efflux from root cells [21] and the same we found in the A.t. mesophyll cells. Transgene *Arabidopsis* overexpressing the SOS1 protein caused an enhanced tolerance to NaCl, since this plant accumulated less Na^+^ in the transpiration stream, and in the shoot, than the wild type [21]. Similar findings were obtained from an *sos1* mutant of rice that showed a higher total uptake of Na^+^ than the wild type [35]. 

A low and transient uptake into the cytosol is more important for tolerance than a total uptake into cells [36]. Here we show that the net uptake of Na^+^ into the cytosol of A.t is higher in the *sos1-1* mutant than in the wild type. It was demonstrated that the *Arabidopsis sos1-1* mutant has reduced growth compared with the wild type, and chlorotic young leaves, which can be expected if higher Na^+^ is present in the cytosol [23]. 

The *nhx1* mutant, lacking the Na^+^/H^+^ antiporter in the tonoplast [8] immediately after NaCl addition, took up less Na^+^ than the other genotypes, however, the net accumulation of Na^+^ was higher than the Wt (Figure 1c and Figure 2). Therefore, this genotype can rapidly transport some Na^+^ out from the cytosol, but not into the vacuole. Thus, our results confirm that AtSOS1-1 is more effective than AtNHX1 in removing Na^+^ from the cytosol of *Arabidopsis* mesophyll cells. 

In experiments with protoplasts from *Arabidopsis*, *Oryza sativa*, and *Vicia faba*, the rise in [Ca^2+^]_cyt_ was lower than in intact transgene *Arabidopsis* subjected to different types of stress [26,27,37,38,39] and might depend on the lack of cell walls. Gao et al. (2004) [38] reported that calcium signalling also could take place in the cell walls. In intact *Arabidopsis* seedlings expressing the calcium–binding protein aequorin, the Ca^2+^_cyt_ elevations were inhibited by La, and to a lesser extent by EGTA [37]. However, lantan and EGTA, which block calcium influx from external stores, were only partly inhibiting the influx of Ca^2+^. Calcium elevation may also result from the activation of PLC, phospholipase C, leading to the hydrolysis of PIP_2_ to IP_3_ and subsequent release of Ca^2+^ from intracellular stores, like the vacuole [40]. Inhibitors to IP_3_ signaling such as LiCl showed that mannitol caused a calcium concentration increase in the microdomain of the vacuole [37]. Our results with the *Arabidopsis* protoplasts from the wild type show that LiCl also blocked the Ca^2+^ elevation caused by NaCl addition. Other findings showed that signalling via phospholipase C was suggested for proline accumulation in *Arabidopsis* upon ionic, but not nonionic hyperosmotic stress [41]. As reported for *Arabidopsis* roots, ROS produced by high salt could activate calcium channels in the tonoplasts [20], and the same mechanism is likely to exist in mesophyll cells.

In the present study we show that NaCl addition, in the presence of 1 mM external Ca^2+^, causes a significant elevation of Ca^2+^_cyt_ in *Arabidopsis* mesophyll protoplasts from the wild type, and a smaller elevation in the *nhx-1* mutant, however, the elevation is lacking in protoplasts from the *sos1-1* genotype (Figure 4). Moreover, in the presence of 0.1 mM Ca^2+^, the calcium increase is much reduced in Wt. Thus, it is likely that Na^+^ at first induces openings of the calcium channels in the plasma membrane and then causes a second calcium elevation from internal stores. Similar results were obtained with a salt-sensitive rice cv. BRRI Dhan29, which also showed a more pronounced elevation at 1.0 mM than at 0.1 mM Ca^2+^ [26]. 

In *Arabidopsis*, the long C-terminal tail of the SOS1 protein located in the cytosol was speculated to be a Na^+^ sensor [5,42,43]. More recent findings suggest that external Na^+^ can bind to a plasma membrane lipid called glycosyl inositol phosphorylceramide, GIPC [44,45]. The Na-bound protein can enter the cytosol and activate an unknown calcium channel leading to an influx of calcium into the cytosol. These findings are in line with the results from two rice cultivars, the salt-tolerant cv. Pokkali, and the salt-sensitive cv. BRRI Dhan 29, which revealed that Na^+^ should enter the cytosol before a [Ca^2+^]_cyt_ elevation could occur [26].

## 4. Materials and Methods

### 4.1. Plant Material

Three genotypes of *Arabidopsis thaliana*, cv. Columbia, wild type, *sos1-1,* and *nhx1*, were obtained from Nottingham Arabidopsis Stock Centre (NASC http://arabidopsis, Loughborough, UK). They were cultivated in a climate chamber in a mixture of vermicompost (Blomjord, Hasselfors Garden, Örebro, Sweden), sand, and plant perlite at a ratio of 2:1:0.5, supplemented with a full-strength nutrient solution (Blomstra, Sweden) once a week during 6–8 weeks [46]. The day/night temperature was 18 ± 2 °C at 18/6 h light/dark photoperiod. The light was a mixture of light from fluorescence tubes L30W/77-Fluora and 30W41-827 LUMILUX; OSRAM, Berlin, Germany). Light intensity was 100 ± 20 µmol s^−1^ m^−2^, and humidity was about 60%.

### 4.2. Protoplast Isolation

Leaves (0.5 g) were sliced in 0.5 mm pieces and digested in darkness with 0.5% (*w*/*v*) cellulase (lyophilized powder; 6 units mg^−1^ solid from *Trichoderma resei* (Sigma, St Louis, MO, USA, EC 3.2.1.4) and 0.2% (*w*/*v*) macerase (lyophilized powder; 0.104 units pectinase mg^−1^), Maceroenzyme R-10, from *Rhizopus* sp. (Serva, Heidelberg, Germany, EC 3.2.1.4) for 1.5 h at 22 ± 1 °C. The pH of the digestion solution (5 mL) was 5.5 and contained 1.0 mM CaCl_2_, 0.2% (*w*/*v*) BSA (Sigma), 0.05% (*w*/*v*) PVP, polyvinyl polypyrrolidone (Sigma) and 20 mM Mes-KOH, [morpholino] ethane sulfonic acid (Sigma). 

After digestion, the solution with plant material was filtrated by a tea strainer and washed twice with the same digestion solution, but without enzymes. Thereafter, it was filtrated through a nylon net (100 µm pores) and centrifuged at 100× *g* for 5 min. The pellet was re-suspended in 1 mL 22% (*w*/*v*) sucrose (BHD, England) and centrifuged at 150× *g* for 5 min. The viable protoplasts floating on the sucrose solution were collected and gently mixed with a washing solution containing 0.5 M mannitol, 1 mM CaCl_2,_ and 20 mM MES-KOH buffer, pH 5.5. The solution was again centrifuged at 100× *g* and the pellet was suspended in 1 mL mannitol solution.

### 4.3. Dye Loading and Fluorescence Measurements

The protoplasts were washed twice in the loading medium (medium ‘A’) containing 0.5 M mannitol (Sigma), 1.0 mM CaCl_2_, 0.05% (*w*/*v*) PVP, 0.2 (*w*/*v*)% BSA (Sigma) and a buffer (pH 5.5) containing 5 mM Tris (Labassco, Partille, Sweden) and 5 mM MES (Sigma), pH 5.5. 

For cytosolic Ca^2+^ measurement, the protoplasts were loaded with acetoxy methyl ester of calcium-binding benzofuran (Fura 2-AM; Molecular Probes, Leiden, the Netherlands). The Fura 2-AM solution was prepared by mixing 2 µL of Fura 2-AM stock solution (5 mg/mL) in dry (<0.1% *v*/*v* water) DMSO, 1.25 µL of pluronic F-127 (Molecular Probes) solution (20% *w*/*v* in DMSO), and 6.75 µL of ethanol (99.5% *v*/*v*) [47,48,49]. From the Fura 2-AM dye solution, 5 µL was added to 1 mL of protoplast suspension (medium A). Loading was performed at 22 °C for 1.5 h.

For Na^+^ measurements protoplasts were loaded with SBFI-AM (Molecular Probes, Eugene, OR, USA) The dye was dissolved in dimethyl sulphoxide (DMSO, Merck, Eurolab AB, Stockholm, Sweden, (<0.1% water) to give a 5 mM stock solution. Two µL of the stock solution was diluted with 6.75 µL ethanol (Kemetyl, Stockholm, Sweden) and 1.25 µL pluronic F-127 (Molecular Probes) as described earlier [31,49] and added to 1 mL of protoplast suspension to get a final concentration of 10 µM. Dye loading was performed in medium A for 1.5–2 h at room temperature in darkness. After loading, the samples were centrifuged and pellets were re-suspended into 1 mL of a solution similar to medium A, but with TRIS-MES buffer at pH 7 (medium B). Before measurements, samples were kept in darkness at room temperature for 25 min.

### 4.4. Fluorescence Measurements

An epi-fluorescence microscope (Axiovert 10; Zeiss, Oberkochem, Germany), supplied with an electromagnetic filter exchanger (Zeiss), Xenon lamp (ZeissXBO 75), photometer (Zeiss 01), microprocessor (MSP 21, Zeiss), and a personal computer was used to determine fluorescence intensity after excitation at 340/380 nm for both 

Fura 2- and SBFI measurements. Measurements were taken every 250 ms. Emission wavelengths were 510–550 nm for both SBFI and Fura 2 measurements. All measurements were performed with a Planneofluar x40/0.75 objective (Zeiss) for phase contrast. Adjustments of signals and noise were made automatically. By means of ratio microscopy, the effect of different dye concentrations can be eliminated [50,51]. Micro slides were covered with poly-L-lysine (molecular weight based on viscosity 150,000–300,000; Sigma) 0.2%, to attach protoplasts to their surface.

Since the *Arabidopsis* protoplasts are very sensitive to osmotic stress, all solutions used had the same osmotic pressure. Thus, when NaCl solutions were added less mannitol was used in the buffer.

In situ calibration for Ca^2+^ measurement was made using single protoplasts labeled with Fura 2-AM as described earlier [51,52].

In situ calibration was also used for [Na.]_cyt_ measurements and made on single protoplasts labeled with SBFI-AM [39]. Measurements of SBFI-fluorescence at an excitation ratio of 340/380 nm and 510–550 nm emission wavelengths were performed with protoplasts in separate suspension solutions with concentrations of 0, 25, 50, 75, and 100 mM NaCl. KCl was added to the suspension solutions to give a final concentration of 100 mM [Na + K] to approximate physiological ionic strengths. The standard measurements were undertaken 5 to 10 min after the addition of 10 mM gramicidin (Sigma) to equilibrate intracellular and extracellular concentrations of Na. As salt stress also induces cytosolic acidification, 5 mM nigericin was added to avoid a pH effect.

### 4.5. Statistics

Each plot is a copy of printer plots and shows representative traces of a specific experiment repeated more than, or equal to, five times with protoplasts from independent cultivations. Each value before salt addition is the average of around 25 fluorescent-ratio determinations and after salt addition, 240 fluorescent-ratio determinations. Figure 3 shows data from experiments repeated five or more times, and Figure 4 from experiments repeated 3 times with protoplasts from independent cultivations. Data are presented as means ± SE. All collected data were statistically analyzed using a factorial completely randomized design (CRD) and the means were compared using the least significant difference test (L.S.D.) at a 5% level of probability to indicate treatment differences [53]

## 5. Conclusions

Our results demonstrate that AtSOS1 under high salinity reduces the Na^+^ concentration in the cytosol of mesophyll cells more effectively than AtNHX1. Similar results were obtained in rice [54]. In *Arabidopsis,* high Na^+^ induces a cytosolic calcium elevation only in cells with an intact SOS1. The calcium elevation is caused by transport from both external and internal stores and is more pronounced in the presence of 1.0 mM external Ca^2+^ than at 0.1 mM. Therefore, it is likely that calcium first enters the cytosol via calcium channels in the plasma membrane probably activated by a Na^+^-GIPC complex [44] and thereafter from internal stores. The AtTPC1 channel in the tonoplast is activated by Ca^2+^_cyt_ and may be involved in calcium transport from the vacuole into the cytosol [48,54].

## Data Availability

Data are available from the corresponding author.

## References

[1] Munns R., Gilliham M. (2015). Salinity tolerance of crops—What is the costs?. New Phytol..

[2] Munns R., James R.A., Läuchli A. (2006). Approaches to increasing the salt tolerance of wheat and other cereals. J. Exp. Bot..

[3] Maathuis F.J.M. (2014). Sodium in plants: Perception, signalling and regulation of sodium fluxes. J. Exp. Bot..

[4] Keisham M., Mukherjee S., Bhatla S. (2018). Mechanisms of sodium transport in plants—Progresses and challenges. Int. J. Mol. Sci..

[5] Zhu J.K. (2003). Regulation of ion homeostasis under salt stress. Curr. Opin. Plant Biol..

[6] Guo Q., Meng L., Han J., Mao P., Tian X., Zheng M., Mur L.A.J. (2020). SOS1 is a key systemic regulator of salt secretion and K^+^/Na^+^ homeostasis in the recretohalophyte *Karelinia caspia*. Environ. J. Exp. Bot..

[7] Pardo J.M., Cubero B., Leidi E.O., Quintero F.J. (2006). Alkali cation exchangers: Roles on cellular homeostasis and stress tolerance. J. Exp. Bot..

[8] Apse M.P., Blumwald E. (2007). Na^+^ transport in plants. FEBS Lett..

[9] Brini F., Hanin M., Mezghani I., Gerald A., Berkowitz G.A., Masmoudi K. (2007). Overexpression of wheat Na^+^/H^+^ antiporter TNHX1 and H^+^-pyrophosphatase TVP1 improve salt- and drought-stress tolerance in *Arabidopsis thaliana* plants. J. Exp. Bot..

[10] Krebs M., Beyhlb D., Esther Görlich E., Al-Rasheid K.A.S., Marten I., Stierhofd Y.-D., Hedrich R., Schumacher K. (2010). *Arabidopsis* V-ATPase activity at the tonoplast is required for efficient nutrient storage but not for sodium accumulation. Proc. Natl. Acad. Sci. USA.

[11] Andres Z., Perez-Hormaeche J., Leidi E.O., Schlucking K., Steinhorst L., McLachlan D.H., Schumacher K., Hetherington A.M., Kudla J., Cubero B. (2014). Control of vacuolar dynamics and regulation of stomatal aperture by tonoplast potassium uptake. Proc. Natl. Acad. Sci. USA.

[12] Luo L., Zhang P., Zhu R., Fu J., Su J., Zheng J., Wang Z., Wang D., Gong Q. (2017). Autophagy is rapidly induced by salt stress and is required for salt tolerance in Arabidopsis. Front. Plant Sci..

[13] Köster P., Wallrad L., Edel K.H., Faisal M., Alatar A.A., Kudla J. (2018). The battles of two ions: Ca^2+^ signalling against Na^+^ stress. Plant Biol..

[14] Wang Q., Guan C., Wang P., Ma Q., Bao A.-K., Zhang J.-L., Wan S.-M. (2019). The Effect of AtHKT1;1 or AtSOS1 mutation on the expressions of Na^+^ or K^+^ transporter genes and ion homeostasis in Arabidopsis thaliana under salt stress. Int. J. Mol. Sci..

[15] Nayef M.A., Celymar S., Shabala L., Ogura T., Chen Z., Bose J., Maathuis F.J.M., Venkataraman G., Tanoi K., Yu M. (2020). Changes in expression level of OsHKT1; 5 alters activity of membrane transporters involved in K^+^ and Ca^2+^ acquisition and homeostasis in salinized rice roots. Int. J. Mol. Sci..

[16] Evans M.J., Choi W.-G., Gilroy S., Morris R.J. (2016). A ROS-assisted calcium wave dependent on the AtRBOHD NADPH oxidase and TPCl cation channel propagates the systemic response to salt stress. Plant Physiol..

[17] White P.J., Broadley M.R. (2003). Calcium in Plants. Ann. Bot..

[18] Park C.-J., Shin R. (2022). Calcium channels and transporters: Roles in response to biotic and abiotic stresses. Front. Plant Sci..

[19] Ye F., Xu L., Li X., Zen W., Gan N., Zhao C., Yang W., Jiang Y., Guo J. (2021). Voltage-gating and cytosolic Ca^2+^ activation mechanisms of Arabidopsis two-pore channel AtTPC1. Proc. Natl. Acad. Sci. USA.

[20] Leshem Y., Melamed-Book N., Cagnac O., Ronen G., Nishri Y., Solomon M., Cohen G., Levine A. (2006). Suppression of *Arabidopsis* vesicle-SNARE expression inhibited fusion of H_2_O_2_-containing vesicles with tonoplast and increased salt tolerance. Proc. Natl. Acad. Sci. USA.

[21] Shi H., Quintero F.J., Pardo J.M., Zhu J.-K. (2002). The Putative plasma membrane Na^+^/H^+^ antiporter SOS1 controls long-distance Na^+^ transports in plants. Plant Cell.

[22] Julkowska M.M., Testerink C. (2015). Tuning plant signaling and growth to survive salt. Trends Plant Sci..

[23] Quintero F.J., Martinez-Atienza J., Villalta I., Jiang X., Kim W.-Y., Ali Z., Fujii H., Mendoza I., Yun D.-J., Zhu J.-K. (2011). Activation of the plasma membrane Na/H antiporter Salt-Overly-Sensitive 1 (SOS1) by phosphorylation of an auto-inhibitory C-terminal domain. Proc. Natl. Acad. Sci. USA.

[24] Quan R., Lin H., Mendoza I., Zhang Y., Cao W., Yang Y., Shang M., Chen S., Pardo J.M., Guo Y. (2007). SCABP8/CBL10, a putative calcium sensor, interacts with the protein kinase SOS2 to protect Arabidopsis shoots from salt stress. Plant Cell.

[25] Egea I., Pined B.A., Ortíz-Atienza A., Plasencia F.A., Drevensek S., García-Sogo B., Yuste-Lisbona F.J., Barrero-Gil J., Atarés A., Flores F.B. (2018). The SlCBL10 Calcineurin B-like protein ensures plant growth under salt stress by regulating Na^+^ and Ca^2+^ homeostasis. Plant Physiol..

[26] Kader A., Lindberg S., Seidel T., Golldack D., Yemelyanov V. (2007). Sodium sensing induces different changes in free cytosolic calcium concentration and pH in salt-tolerant and salt-sensitive rice (*Oryza sativa* L.) cultivars. Physiol. Plant..

[27] Morgan S.H., Lindberg S., Jha Maity P., Geilfus C.M., Plieth C., Mühling K.H. (2017). Apoplastic and cytosolic Ca^2+^ and pH dynamics in salt-stressed Vicia faba leaves change in response to calcium. Func. Plant Biol..

[28] Villalobos-Lo’pez M.A., Arroyo-Becerra A., Quintero-Jiménez A., Iturriaga G. (2022). Biotechnological Advances to Improve Abiotic Stress Tolerance in Crops. Int. J. Mol. Sci..

[29] Ding L., Zhu J.-K. (1997). Reduced Na^+^ uptake in the NaCI-hypersensitive sos1 mutant of Arabidopsis thaliana. Plant Physiol..

[30] Essah P.A., Davenport R., Tester M. (2003). Sodium influx and accumulation in Arabidopsis. Plant Physiol..

[31] Kader M.A., Lindberg S. (2005). Uptake of sodium in protoplasts of salt-sensitive and salt-tolerant cultivars of rice, *Oryza sativa* L. determined by the fluorescent dye SBFI. J. Exp. Bot..

[32] Tada Y., Ohnuma A. (2020). Comparative Functional Analysis of Class II Potassium Transporters, SvHKT2;1, SvHKT2;2, and HvHKT2;1, on Ionic Transport and Salt Tolerance in Transgenic Arabidopsis. Plants.

[33] Yemelyanov V.V., Shishova M.F., Chirkova T.V., Lindberg S. (2011). Anoxia-induced elevation of cytosolic Ca^2+^ concentration depends on different Ca^2+^ sources in rice and wheat protoplasts. Planta.

[34] Demidchik V., Maathuis F.J.M. (2007). Physiological roles of nonselective cation channels in plants: From salt stress to signalling and development. New Phytol..

[35] El Mahi H., Péres-Hormaeche J., De Luca A., Villlalta I., Espartero J., Gámes-Arjona F., Fernández J.L., Bundó M., Mendoza I., Mieulet D. (2019). A Critical Role of Sodium Flux via the Plasma Membrane Na+/H+ Exchanger SOS1 in the Salt Tolerance of Rice. Plant Physiol..

[36] D’Onofrio C., Kader A., Lindberg S. (2005). Uptake of sodium in quince, wheat and sugar beet protoplasts determined by the fluorescent sodium-binding benzofuran isophthalate dye. J. Plant Physiol..

[37] Knight H., Trewavas A.J., Knight M.R. (1997). Calcium signal. Plant J..

[38] Gao D., Knight M.R., Trewavas A.J., Sattelmacher B., Plieth C. (2004). Self-reporting Arabidopsis expressing pH and [Ca^2+^] indicators unveil ion dynamics in the cytoplasm and in the apoplast under abiotic stress. Plant Physiol..

[39] Morgan S.H., Geilfus C.M., Lindberg S., Mühling K.H. (2014). The leaf ion homeostasis and PM H^+^-ATPase activity in *Vicia faba* L. change after extra calcium and potassium supply under salinity. Plant Physiol. Biochem..

[40] Allen G.J., Muir S.R., Sanders D. (1995). Release of Ca^2+^ from individual plant vacuoles by both InsP3 and cyclic ADP-ribose. Science.

[41] Parre E., Ghars M.A., Leprince A.-S., Thiery L., Lefebvre D., Bordenave M., Richard L., Mazars C., Abdelly C., Savouré A. (2007). Calcium signaling via phospholipase C is essential for proline accumulation upon ionic but not nonionic hyperosmotic stresses in Arabidopsis. Plant Physiol..

[42] Zhang J.Z., Creelman R.A., Zhu J.-K. (2004). From laboratory to field. Using information from Arabidopsis to engineer salt, cold, and drought tolerance in crops. Plant Physiol..

[43] Shabala L., Cuin T.A., Newman I.A., Shabala S. (2005). Salinity-induced ion flux patterns from the excised roots of Arabidopsis *sos* mutants. Planta.

[44] Jiang Z., Zhou X., Pei Z.-M. (2019). Plant cell-surface GIPC sphingolipids sense salt to trigger Ca^2+^ influx. Nature.

[45] Steinhorst L., Kudla J. (2019). How plants perceive salt. Nature.

[46] Premkumar A., Lindberg S., Lager I., Rasmussen U., Schulz A. (2019). Arabidopsis PLDs with C2-domain function distinctively in hypoxia signaling. Physiol. Plant..

[47] Sebastiani L., Lindberg S., Vitagliano C. (1999). Cytoplasmic free calcium dynamics in single tomato (*Lycopersicon esculentum* L) protoplasts subjected to chilling temperatures. Physiol. Plant..

[48] Poenie M., Alderton J., Steinhardt R., Tsien R. (1986). Calcium rises abruptly and briefly throughout the cell cycle and onset of anaphase. Science.

[49] Bright G.R., Fisher G.W., Rogowska J., Taylor D.L. (1987). Fluorescence ratio imaging microscopy: Temporal and spatial measurements of cytoplasmic pH. J. Cell Biol..

[50] Tsien R.Y., Poenie M. (1986). Fluorescence ratio imaging: A new window into intracellular ionic signalling. Trends Biochem. Sci..

[51] Lindberg S., Premkumar A., Rasmussen U., Schulz A., Lager I. (2018). Phospholipases AtPLDζ1 and AtPLDζ2 function differently in hypoxia. Physiol. Plant..

[52] Grynkiewicz G., Poenie M., Tsien R.Y. (1985). A new generation of Ca^2+^ indicators with greatly improved fluorescence properties. J. Biol. Chem..

[53] Snedecor G.W., Cochran W.G. (1980). Statistical Methods.

[54] Schulze C., Sticht H., Meyerhoff P., Dietrich P. (2011). Differential contribution of EF-hands to the Ca^2+^-dependent activation in the plant two-pore channel TPC1. Plant J..

